# Transcriptomic analysis reveals insights into the response to *Hop stunt viroid* (HSVd) in sweet cherry (*Prunus avium* L.) fruits

**DOI:** 10.7717/peerj.10005

**Published:** 2020-09-21

**Authors:** Li Xu, Xiaojuan Zong, Jiawei Wang, Hairong Wei, Xin Chen, Qingzhong Liu

**Affiliations:** Key Laboratory for Fruit Biotechnology Breeding of Shandong Province, Shandong Institute of Pomology, Taian, Shandong, People’s Republic of China

**Keywords:** Sweet cherry, Hop stunt viroid, Transcriptome, RT-qPCR

## Abstract

*Hop stunt viroid* (HSVd) is a member of the genus *Hostuviroid* of the family Pospiviroidae and has been found in a wide range of herbaceous and woody hosts. It causes serious dapple fruit symptoms on infected sweet cherry, notably inducing cherry tree decay. In order to better understand the molecular mechanisms of HSVd infection in sweet cherry fruit, transcriptome analysis of HSVd-infected and healthy sweet cherry fruits was carried out. A total of 1,572 differentially expressed genes (DEGs) were identified, involving 961 upregulated DEGs and 611 downregulated DEGs. Functional analysis indicated that the DEGs were mainly involved in plant hormone signal transduction, plant–pathogen interactions, secondary metabolism, and the MAPK signaling pathway. In addition, C2H2 zinc finger, MYB, bHLH, AP2/ERF, C2C2-dof, NAC and WRKY transcription factors can respond to HSVd infection. In order to confirm the high-throughput sequencing results, 16 DEGs were verified by RT-qPCR analysis. The results provided insight into the pathways and genes of sweet cherry fruit in response to HSVd infection.

## Introduction

Viroids are circular, single-stranded, non-protein-coding infectious RNAs with a genome range of 246–401 nucleotide (Hadidi, 2019). They are economically important because they systematically replicate and spread when they infect susceptible plant hosts. The potato spindle tuber disease is caused by the first viroid discovered (Diener, 1971a, 1971b). Viroid species are divided into Pospiviroidae and Avsunviroidae based on the presence or absence of the conserved central region (CCR) in their genome (Di Serio et al., 2014). *Hop stunt viroid* (HSVd) is the type member of genus *Hostuviroid* of the family Pospiviroidae, which is widely distributed in orchard all over the world. HSVd was first discovered in Japanese hop fields, causing hop stunt disease (Yamamoto et al., 1973; Kappagantu et al., 2017b). Following this, it was reported in plum, peach, citrus, cucumber, apricot, almond and sweet cherry, etc., where it induced dappled fruit, cachexia of citrus, pale fruits of cucumber and fruit degeneration of apricot (Sano et al., 1989; Xu et al., 2017; Semancik et al., 1988; Diener et al., 1988; Lemmetty, Werkman & Soukainen, 2011; Amari et al., 2007). Symptoms varied from mild to severe and could affect either the entire plant or specific organs.

Sweet cherry (*Prunus avium* L.) is one of the most popular and economically valuable fruits. It was introduced into China in 1871 and is now the most cultivated *Prunus* species in China. The total plantation area of cherries reached approximately 266,000 hm^2^ in 2018, including sweet cherry, accounting for 233,000 hm^2^ and Chinese cherry, accounting for 33,000 hm^2^, with a total production of 1,700,000 tons (Duan et al., 2019). HSVd infection produces severe symptoms in sweet cherry fruits, leading to unsaleable fruits and severe economic losses. It is well known that the skin of fleshy fruit has high economic relevance for fruit quality. The fruit’s soluble solids concentration and visual skin color influence consumer acceptance of cherries (Crisosto, Crisosto & Metheney, 2003). Therefore, it is very important to understand the molecular mechanisms of HSVd pathogenesis and further development of HSVd management strategies.

High-throughput RNA-Seq techniques have been widely used to study the transcriptome changes of plants infected by pathogens. Several viroid–plant interaction analyses have been analyzed at the transcriptional level to study changes in both the physiological and metabolic processes of infected plants (Katsarou et al., 2016; Zheng et al., 2017; Kappagantu et al., 2017a). Xia et al. (2017) employed HSVd-infected cucumber (*Cucumis sativus* cv. “Suyo”) as a model system in which to examine genome-wide changes in gene expression using RNA-Seq. Wang et al. (2019) demonstrated that basic defense and RNA-silencing mechanisms were activated by *Citrus exocortis viroid* (CEVd) infection in “Etrog” citron. Mishra et al. (2018) employed comprehensive transcriptome analyses, which revealed that *Citrus bark cracking viroid* (CBCVd) infection influenced the expression of more than 2,000 genes, which could be associated with the systemic symptom development. Štajner et al. (2019) performed a comparative transcriptomic analysis and indicated the transcriptional changes caused by single *Hop Latent Viroid* (HLVd) and CBCVd infection in hop. These studies suggested that genes involved in secondary metabolism, plant immune responses, phytohormone signaling pathways, photosynthesis, and other processes, were more strongly modulated upon viroid infection. The purpose of our study is to investigate transcriptional variations in sweet cherry fruits upon HSVd infection.

In this study, we conduct a comparative analysis of transcriptome profiles between HSVd-infected and healthy sweet cherry fruits. This is the first transcriptome study to identify differentially expressed genes (DEGs) in HSVd-infected sweet cherry fruits. Genes associated with plant–pathogen interactions, hormone signal transduction, secondary metabolism, and the MAPK signaling pathway showed differential expression. We also found that phenylpropanoid biosynthesis and flavonoid biosynthesis pathways might play key roles in dapple fruit formation upon HSVd infection. These results will lay a good foundation for future research on the interaction between plants and viroids, and will further promote the development of effective measures for the prevention and treatment of woody plant viroids.

## Materials and Methods

### Plant material

All fruits were collected from 10-year-old, field-grown sweet cherry (*Prunus avium* L.) cultivar “hongdeng” in our own orchard in Taian, Shandong Province, China. Fruit samples were collected from HSVd-infected and healthy trees. Six individual fruits from three trees were pooled as one sample from each group, and three replicates were used for RNA-seq and RT‑qPCR in our study. The collecting date was 30 day after full bloom (DAFB). The peel of fruits were immediately frozen in liquid nitrogen and stored at −80 °C until further use.

### RNA extraction, library construction and illumina sequencing

Total RNA was extracted using the total RNA kit (Tiangen, Beijing, China) according to the manufacturer’s protocol. RNA samples were treated with RNase-free DNase I (Takara, Dalian, China) to avoid DNA contamination. Agarose gel electrophoresis (1.5%), a Nanodrop 2000 (Thermo Scientific, Wilmington, NC, USA), and an Agilent 2100 Bioanalyzer (Agilent Technologies, Santa Clara, CA, USA) were used to check the integrity, concentration, and purity of RNA. Library preparation was performed using the NEBNext^®^ Ultra^™^ RNALibrary Prep Kit for Illumina^®^ (NEB, Ipswich, MA, USA) following the manufacturer’s instructions. The libraries were sequenced on an Illumina HiSeq 2000 platform at the Novogene Bioinformatics Technology Co., Ltd. (Beijing, China). All raw data were submitted to the National Center for Biotechnology Information (NCBI) Sequence Read Archive (SRA) (No. PRJNA596893).

### Data analysis of transcriptome sequencing

Clean reads were obtained by removing reads with only an adaptor or those with low quality from the raw data. Additionally, reads less than 50 nt were eliminated. Then, 150 bp of paired-end reads were generated. The Q20, Q30, GC content, and sequence duplication level of the clean data were calculated. All downstream analyses were based on clean data of a high quality. The sweet cherry (*Prunus avium* L.) (assembly PAV_r1.0) genomic sequences were used as a reference (https://www.ncbi.nlm.nih.gov/genome/11780). The Bowtie software (v2.1.0) was used for read mapping and assembly. The gene expression was determined as RPKM (reads per kilobase of exon model per million mapped reads). Differential expression analysis of HSVd-infected and healthy fruits was performed with the R-based package DESeq. DEGs were calculated based on log_2_ of the fold change (log_2_ FC), and |log_2_ FC| ≥ 2 were considered to be differentially expressed.

### GO and KEGG enrichment analyses of DEGs

To determine the functional annotation of DEGs, BLAST alignment was used to search against the GO and KEGG databases and retrieve protein functional annotations based on sequence similarity. GO enrichment (*p*-value < 0.05) analysis of the DEGs was implemented by GOseq R packages based on Wallenius non-central hyper-geometric distribution, which could be adjusted for gene length bias in DEGs. KEGG is a knowledge base for systematic analysis of gene functions in terms of the networks of genes and molecules. KEGG terms with a corrected *p*-value < 0.05 were considered to be significantly enriched in DEGs.

### Prediction of transcription factors

To identify transcription factors expressed in HSVd-infected sweet cherry fruit, all DEGs were searched against the plant TF database (https://plantgrn.noble.org/PlantTFcat/) (Dai et al., 2013) which resulted in the classification of sweet cherry transcripts into various TF families.

### RT‑qPCR validation of RNA sequencing

To validate the differential expression identified by RNA sequencing, RT‑qPCR was performed. The total RNA was extracted following the aforementioned method and was then reverse transcribed with the PrimeScript RT Reagent Kit with gDNA Eraser (Takara, Dalian, China), following the user’s manual. Specific primers for randomly selected genes were designed with Primer3 web version 4.0.0, and amplified PCR products varied from 80 to 150 bp (Table S1). The sweet cherry housekeeping gene β-*actin* was selected as the internal control to calibrate the cDNA template of the corresponding samples (Zong et al., 2015). RT-qPCR was performed using an ABI 7500 Fast Real-Time PCR System (Applied Biosystems, Foster City, CA, USA). The reaction mixture included template cDNA (1 μL), PCR primers (10 μM, 0.4 μL), and 2 × AceQ Universal SYBR qPCR Master Mix (Vazyme Biotech Co.,Ltd., Nanjing, China) in a total volume of 20 μL. The PCR reaction conditions was initiated by hot start at 95 °C for 5 min, followed by 40 cycles at 95 °C for 10 s and 60 °C for 30 s. Three biological replicates were generated, and three measurements were performed on each replicate. The relative expression of the genes was calculated using the 2^−∆∆Ct^ method (Livak & Schmittgen, 2001).

## Results

### Transcriptome sequencing in response to HSVd infection

**Figure 1:**
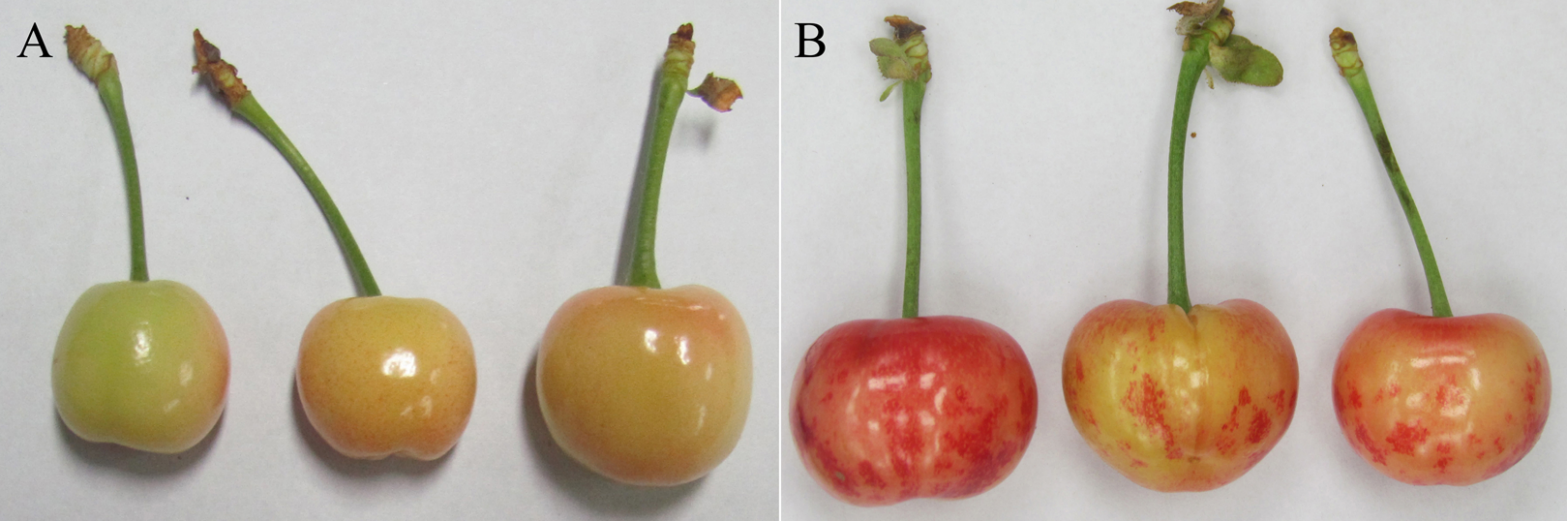
Healthy (A) and HSVd infected (B) sweet cherry fruits.

To investigate the molecular changes that took place in HSVd-infected sweet cherry fruits, cDNA libraries were constructed from the total RNA of “hongdeng” sweet cherry. The sweet cherry samples were green healthy fruits (GHF) (Fig. 1A) and green dapple fruit (GDF) (Fig. 1B). An Illumina deep-sequencing run generated 45.08~67.40 million raw reads per sample. After removal of adaptor sequences and low-quality reads, 43.32~64.67 million clean reads were obtained per sample. The ratio of the Q20 sequencing value was greater than 96%, which indicated that the results were high quality reliable gene transcript data (Table 1). These were then mapped to the sweet cherry reference genome. Which assembled sequences was 272.4 Mb in length, covering 77.8% of the 352.9 Mb sweet cherry genome and included >96.0% of the core eukaryotic genes. 43,349 complete and partial protein-encoding genes were predicted (Shirasawa et al., 2017). In this study, over 88.35% of the reads were mapped to the sweet cherry genome (Table 1). All the sequencing data have been submitted to the Sequence Read Archive (SRA). The accession number is PRJNA596893.

**Table 1:** Statistics of RNA-seq analysis of sweet cherry fruits.

Sample name	Raw reads	Clean reads	Clean bases	RNA-seq error rate (%)	Q20 (%)	Q30 (%)	GC content (%)	Total mapped (%)
Green healthy fruit (GHF1)	54968392	52822456	7.92G	0.02	96.62	91.77	46.75	88.66
Green healthy fruit (GHF2)	57447720	55155662	8.27G	0.02	96.79	92.21	46.79	88.35
Green healthy fruit (GHF3)	45087756	43325014	6.5G	0.02	96.62	91.83	46.65	88.82
Green dapple fruit (GDF1)	59595422	57441392	8.62G	0.02	96.69	92.02	47.49	90.2
Green dapple fruit (GDF2)	47655902	45788010	6.87G	0.02	96.77	92.17	47.47	89.8
Green dapple fruit (GDF3)	67400146	64675352	9.7G	0.02	96.75	92.12	47.43	90.2

### Identification and functional classification of DEGs

**Figure 2:**
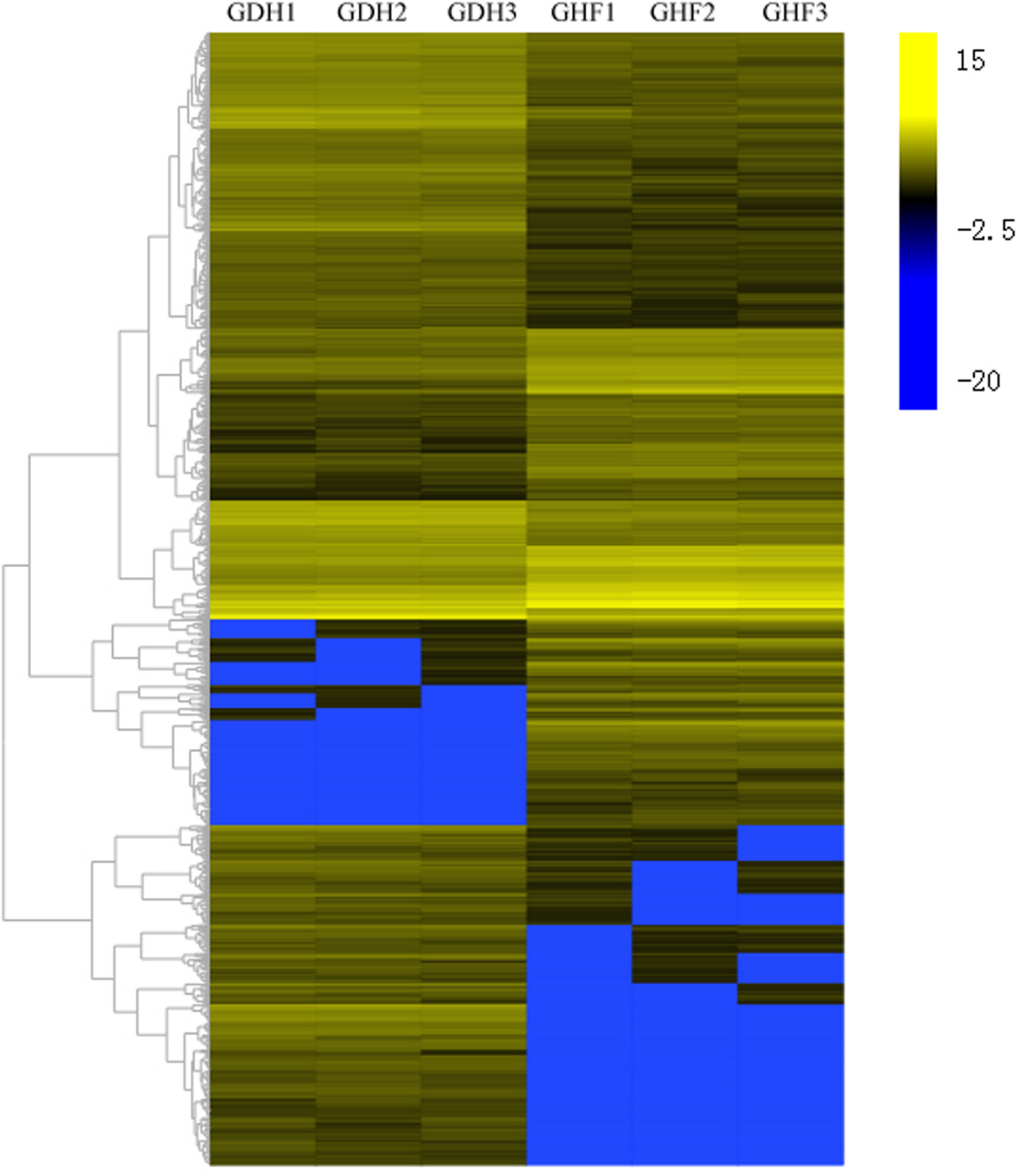
Transcriptome analysis of DEGs. Heat map and complete linkage hierarchical clustering of log_2_ fold change of DEGs between HSVd infected (GDF) and healthy (GHF) sweet cherry fruits. Blue: gene transcript levels decreased; Dark: gene transcript levels is stable; yellow: gene transcript levels increased.

To investigate the DEGs that were associated with HSVd infection, the DEGs were selected according to the corrected *p*-value < 0.05 and |log_2_ FC| ≥ 2. Comparisons were performed between GDF and GHF. A total of the 1,572 genes with significantly differentially expression levels between GDF and GHF, 961 and 611 were up-and down-regulated, respectively (Fig. 2; Table S2), which suggested the significant impact of HSVd infection on host gene expression in sweet cherry fruits. We used the gene cluster set to generate a tree that showed the similarities in the relative gene expressions (Fig. 2).

We performed Gene Ontology (GO) enrichment assignments to analyze the function classification of DEGs in response to HSVd infection. The DEGs were divided into “molecular function”, “cellular component” and “biological process” (Table S3). In the “biological process” category, most genes were functionally assigned into “metabolic process” (335, 21.31%), “cellular process” (264, 16.79%), “single-organism process” (221, 14.06%), “biological regulation” (121, 7.70%) and “response to stimulus” (112, 7.12%). In “molecular function”, the majority of genes were subcategorized into “binding” (383, 24.36%), “catalytic activity” (380, 24.17%) and “transporter activity” (63, 4.01%). In “cellular component”, “membrane” (278, 17.68%), “membrane part” (237, 15.08%), “cell” (195, 12.40%), “cell part” (191, 12.15%) and “organelle” (120, 7.63%) represented the major groups of genes. GO functional enrichment analysis showed the statistically significant and related GO terms of DEGs in HSVd-infected sweet cherry fruits. A total of 21 statistically enriched GO terms during infection of HSVd compared to the whole transcriptome background (corrected *p*-value < 0.05) were screened (Table 2). In the category ‘biological process”, the GO terms linked to “response to stimuli”, “multi-organism process”, “reproduction”, “cellular process” and “multicellular organismal process” were significantly enriched. Similarly, in the category “molecular function’’, GO terms related to “catalytic’’ and “binding activity”, which played crucial roles in signal recognition and transduction, were significantly enrichment. In addition, analysis of the category “cell component” indicated no enriched terms.

**Table 2:** Enriched GO terms for the DEGs in HSVd-infected sweet cherry fruits.

GO ID	Description	Category	Corrected *p*-value
GO:0043531	MF	ADP binding	0.00000000251
GO:0009733	BP	Response to auxin	0.0000000521
GO:0020037	MF	Heme binding	0.0000000997
GO:0046906	MF	Tetrapyrrole binding	0.0000000997
GO:0016705	MF	Oxidoreductase activity, acting on paired donors, with incorporation or reduction of molecular oxygen	0.00000275
GO:0005506	MF	Iron ion binding	0.00000463
GO:0009719	BP	Response to endogenous stimulus	0.00000935
GO:0009725	BP	Response to hormone	0.00000935
GO:0010033	BP	Response to organic substance	0.00000935
GO:0042221	BP	Response to chemical	0.000708
GO:0051704	BP	Multi-organism process	0.007538
GO:0008037	BP	Cell recognition	0.011162
GO:0009856	BP	Pollination	0.011162
GO:0009875	BP	Pollen-pistil interaction	0.011162
GO:0044706	BP	Multi-multicellular organism process	0.011162
GO:0048544	BP	Recognition of pollen	0.011162
GO:0000003	BP	Reproduction	0.013435
GO:0022414	BP	Reproductive process	0.013435
GO:0044702	BP	Single organism reproductive process	0.013435
GO:0030246	MF	Carbohydrate binding	0.030997
GO:0032501	BP	Multicellular organismal process	0.038703

**Note:**

BP, biological process; MF, molecular function.

**Figure 3:**
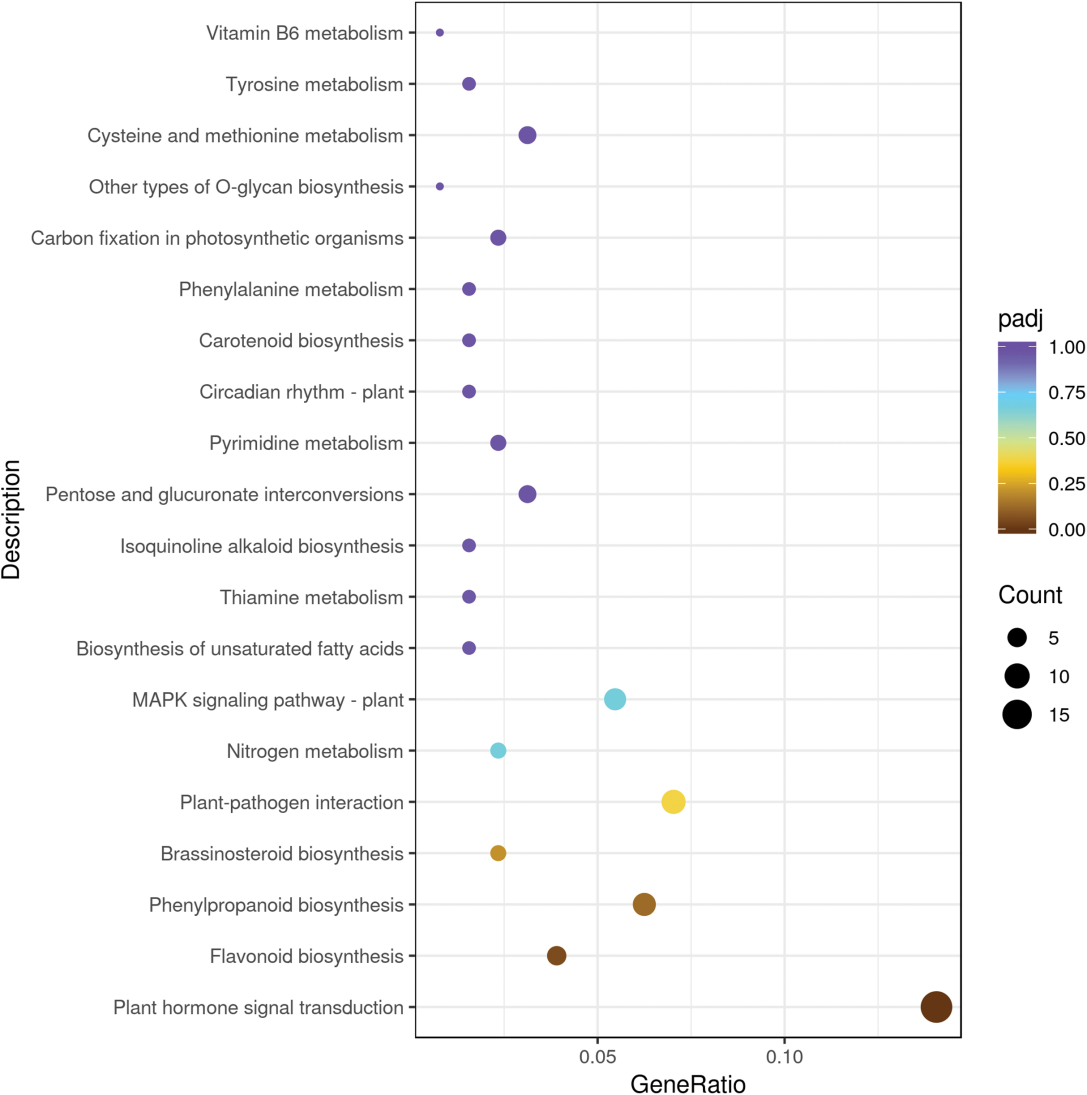
The top 20 pathways enriched for differentially expressed genes. The size of each circle represents the number of significantly differentially expressed genes (up-regulated and down-regulated) enriched in the corresponding pathway.

We mapped the DEGs to KEGG database and seventy KEGG pathways were found in sweet cherry fruits infected by HSVd (Table S4). Metabolism involved the largest number of DEGs, mainly including “lipid metabolism”, “carbohydrate metabolism”, “amino acid metabolism”, “biosynthesis of other secondary metabolites”, “energy metabolism”, and other sub-categories. In “secondary metabolism” categories, the mainly represented subcategories were “prenylflavonoids biosynthesis, “flavonoid biosynthesis”, “isoquinoline alkaloid biosynthesis” and “tropane, piperidine and pyridine alkaloid biosynthesis”. The “genetic information processing” was mainly composed of “folding, sorting and degradation”, followed by “translation” and “replication and repair”. The “environmental information processing” included “signal transduction” such as “plant hormone signal transduction”, “MAPK signaling pathway” and “phosphatidylinositol signaling system” (Table 3). The top 20 significant enrichment pathways were shown in Fig. 3. Among them, the plant hormone signal transduction pathway was the largest group (18 DEGs), followed by the plant–pathogen interaction pathway (nine DEGs), prenylflavonoids biosynthesis (eight DEGs), MAPK signaling pathway (seven DEGs), and flavonoid biosynthesis (five DEGs) (Fig. 3).

**Table 3:** Classification statistics for DEGs of up-regulated (UR) and down-regulated genes (DR) in HSVd-infected sweet cherry fruits according to KEGG pathway analysis.

KEGG categories	Number of		KEGG Categories	Number of	
	UR	DR		UR	DR
Metabolism					
Biosynthesis of other secondary metabolites	14	2	Carbohydrate metabolism	21	9
Metabolism of terpenoids and polyketides	4	1	Nucleotide metabolism	4	2
Energy metabolism	6	1	Amino acid metabolism	8	6
Lipid metabolism	11	4	Glycan biosynthesis and metabolism	0	1
Metabolism of cofactors and vitamins	3	1	Metabolism of other amino acids	2	1
Environmental Information Processing			Organismal Systems		
Signal transduction	20	6	Environmental adaptation	9	2
Genetic Information Processing			Unclassified		
Replication and repair	3	0	Fatty acid metabolism	1	1
Transcription	2	0	Biosynthesis of amino acids	3	2
Folding, sorting and degradation	3	5	2-Oxocarboxylic acid metabolism	1	0
Translation	3	2			
Cellular Processes					
Transport and catabolism	5	2			
			Total	123	48

### HSVd infection impacts the plant hormone signal transduction

Many studies have shown that plant hormones play remarkable roles in the plant life cycle and act as an important regulator of plant growth, development and abiotic/biotic stress responses (Owens et al., 2012). In this study, the plant hormone signal transduction pathway contained the largest number of DEGs. Eighteen DEGs were involved in jasmonic acid (JA), indole-3-acetic acid (IAA), ethylene (ET), abscisic acid (ABA) and salicylic acid (SA) signal transduction pathways under HSVd infection (Table S5).

In the JA signaling pathway, the dissociation between JAZ (jasmonate ZIM domain-containing protein) and the transcription factor MYC2 could induce defense-related genes and was initiated by JA. In this study, one gene (*Pav_sc0001580.1_g140.1.mk*) encoding the JAZ protein was upregulated. For the SA-mediated signal transduction pathway, one gene (*Novel00046*) encoding TGA was upregulated, which could activate the activity of PR-1 (pathogenesis-related protein 1) that is known to be a marker gene for SA. In the ABA and ET signaling pathways, one transcript of protein phosphatase 2C (PP2C) (Pav_sc0002858.1_g200.1.mk) and ethylene-insensitive protein 2 isoform X1 (EIN2) (Pav_co4041309.1_g010.1.br) were significantly upregulated. Auxin response factor*s* (*ARFs*), Gretchen Hagen3s (*GH3s*), auxin/indole-3-acetic acids (*Aux/IAAs*), and small auxin upregulated RNAs (*SAURs*), which have been identified as main early auxin responsive genes and play an important role in auxin signaling and homeostasis, regulate downstream auxin late-response genes in the transcriptional network, and further mediate auxin-related diverse physiological processes (Weijers & Friml, 2009; Hagen & Guilfoyle, 2002). We identified 14 genes associated with auxin metabolism and signaling that were significantly differentially expressed. The genes of *Aux1* and *GH3* were upregulated, and the *Aux/IAA* gene was downregulated. Moreover, six of the homologs of SAUR-like auxin-responsive protein (*SAUR*) were upregulated and five were downregulated.

### HSVd infection impacts the basal defense responses

Plants have evolved different kinds of defense responses to prevent or limit disease. Upon pathogen infection, the activation of the plant defense response is often associated with ion exchange, the generation of reactive oxygen species (ROS), and cell wall reinforcement (Zaynab et al., 2018). Based on the interaction of plant and pathogen, the ROS and oxide (NO) signaling pathways was regulated by the hyper-sensitive response (HR). ROS can be activated by flagellin-sensitive 2 (FLS2) and respiratory burst oxidase homolog (RBOH). In the present study, nine DEGs were identified as participating in the plant–pathogen interaction pathway (Table S6). Among them, two genes (*Pav_sc0000138.1_g1220.1.mk* and *Pav_sc0000480.1_g090.1.mk*) related to the cyclic nucleotide-gated ion channel (CNGC; one up-regulated, one down-regulated) and two genes (*Pav_sc0001911.1_g560.1.mk* and *Pav_sc0001524.1_g050.1.mk*) encoding the disease resistance protein (RPM; one upregulated, one downregulated) were observed. In addition, one upregulated plant calcium-dependent protein kinase (CDPKs) (Pav_sc0000440.1_g200.1.mk) was observed. Further, one upregulated RBOH (Pav_sc0000886.1_g850.1.mk) functioned as an NADPH oxidase to generate ROS, and three transcripts (Pav_sc0000071.1_g810.1.mk, Pav_sc0000484.1_g710.1.mk and Pav_sc0000348.1_g1020.1.mk) related to calcium-binding proteins and calmodulin-like proteins (CaM, CMLs; the three upregulated) were highly expressed.

In the pathogen-triggered immunity (PTI) and effector-triggered immunity (ETI) pathways, the mitogen-activated protein kinase (MAPK) cascade is a central component of signal transduction. Increased transcript levels of genes encoding MAPK have been demonstrated, implying the involving of a MAP kinase pathway in the HSVd stress response. Our RNA-seq results showed that seven DEGs functioning in the MAPK-signaling pathway were significantly up-regulated, i.e., mitogen-activated protein kinase 2 (MAP3K2) (Pav_sc0001077.1_g280.1.mk), calmodulin-like gene (*CaM4*) (*Pav_sc0000484.1_g710.1.mk*), the respiratory burst oxidase homolog (RBOH) (Pav_sc0000886.1_g850.1.mk), protein phosphatase 2C (PP2C) (Pav_sc0002858.1_g200.1.mk), copper-transporting ATPase HMA5 (HMA) (Pav_sc0000599.1_g120.1.mk), the ethylene-insensitive protein (EIN2) (Pav_co4041309.1_g010.1.br) and 1-aminocyclopropane-1-carboxylate synthase (ACS6) (Pav_sc0000886.1_g690.1.mk). These DEGs may play crucial roles in responses to HSVd infection.

### HSVd infection impacts the secondary metabolism

It is well known that plant secondary metabolites are are essential for plant growth and development, and they can also act as defense molecules to protect plants in all kinds of adverse conditions. In the present study, many DEGs were involved in phenylpropanoid biosynthesis and flavonoid biosynthesis (Table S7). The phenylpropanoid pathway was usually activated by pathogen infection, resulting in the production of both phytoalexins and lignin/suberin precursors for cell wall strengthening (Parker et al., 2009). In the phenylpropanoid biosynthetic pathway, one gene (*Pav_co4071347.1_g010.1.mk*) annotated as phenylalanine ammonia lyase (PAL) was upregulated. One gene (*CYP98A*) (*Pav_sc0001196.1_g1100.1.mk*) encoding 5-O-(4-coumaroyl)-D-quinate 3′-monooxygenase and one gene (*HST*) (*Pav_sc0003326.1_g220.1.mk*) encoding shikimate O-hydroxycinnamoyltransferase were up-regulated. One probable gene (*Pav_sc0000729.1_g240.1.br*) encoding 8-hydroxygeraniol dehydrogenase was up-regulated. Three peroxidases (Pav_sc0000028.1_g820.1.mk, Pav_sc0001323.1_g1090.1.mk and Pav_sc0000099.1_g130.1.mk) were upregulated, and one peroxidase (Pav_sc0000017.1_g020.1.mk) was downregulated. Flavonoids constitute a large group of phenolic secondary metabolites and have been shown to have diverse biological functions (Falcone Ferreyra, Rius & Casati, 2012). Homology searches identified five genes that were related to the flavonoid biosynthesis pathway in this study. The transcript levels of chalcone synthase (CHS) (Pav_sc0000045.1_g280.1.mk) and flavonol synthase (FLS) (Pav_sc0000030.1_g1340.1.mk) were upregulated. One gene (*HST*) (*Pav_sc0003326.1_g220.1.mk*) encoding shikimate O-hydroxycinnamoyltransferase and one gene (*CYP98A*) (*Pav_sc0001196.1_g1100.1.mk*) encoding 5-O-(4-coumaroyl)-D-quinate 3’-monooxygenase were upregulated, while one chalcone isomerase gene (*CHI*) (*Pav_sc0007510.1_g020.1.mk*) was downregulated.

### TFs involved in HSVd infection

**Figure 4:**
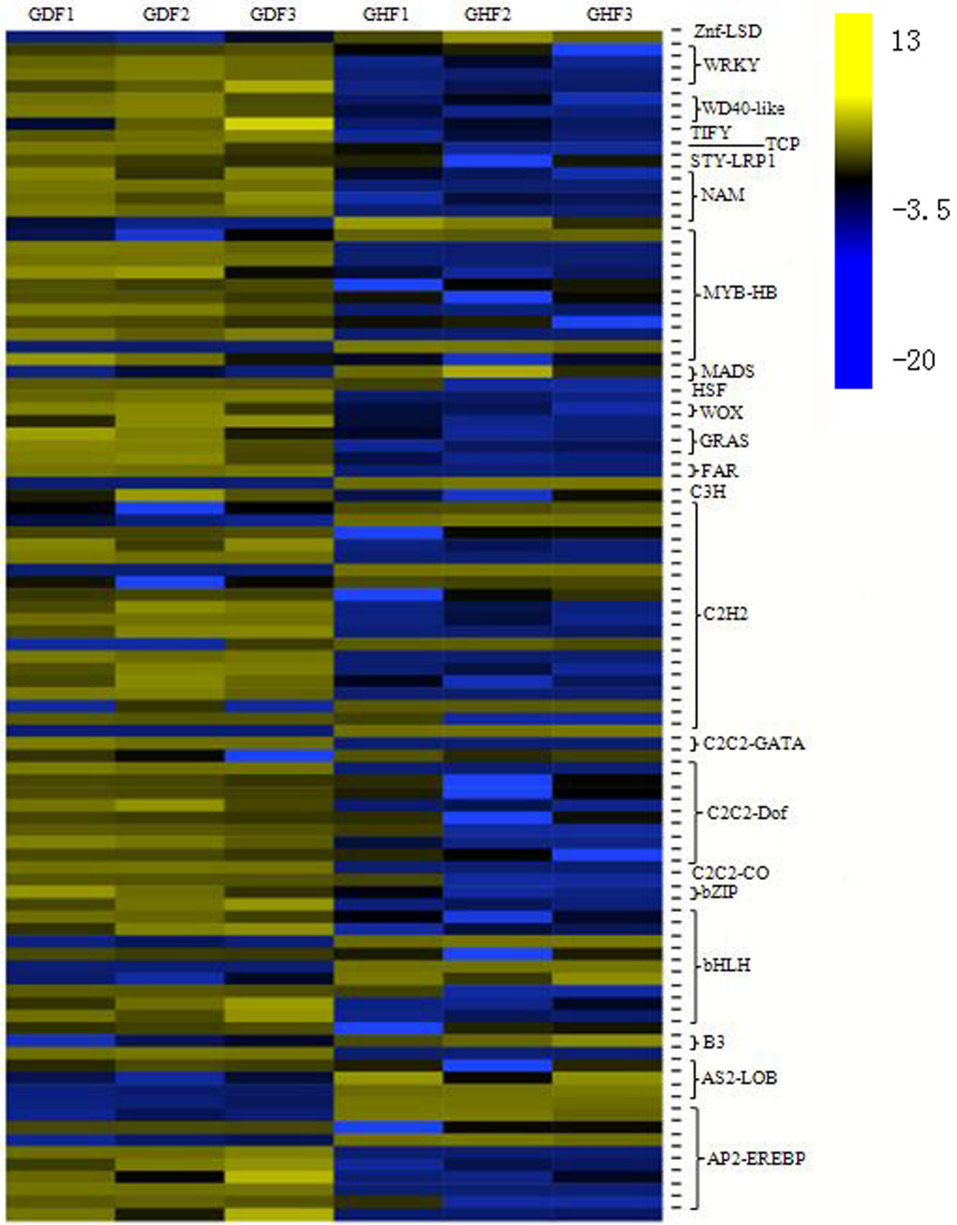
Transcription factors (TFs) involved in response to HSVd infection in sweet cherry fruits. Heat map of TFs between HSVd infected (GDF) and healthy (GHF) sweet cherry fruits. Blue: gene transcript levels decreased; Dark: gene transcript levels is stable; yellow: gene transcript levels increased.

Transcriptional reprograming is one of the significant events in the plant defense response and biochemical and physiological changes principally governed by transcription factors (TFs) (Moore, Loake & Spoel, 2011). More and more studies indicated that several families of TFs play an important role in the regulation of the expression of the plant defense transcriptome (Singh, Foley & Oñate-Sánchez, 2002; Tsuda & Somssich, 2015). In this study, analysis of all DEGs uncovered 96 transcription factors, which were further divided into 23 gene families (Table S8). The C2H2 zinc finger family had the highest number of DEGs (19 genes), followed by the MYB family (11 genes), bHLH family (10 genes), AP2/ERF family (nine genes), C2C2-Dof family (nine genes), NAC family (five genes) and WRKY family (four genes). Based on the expression patterns of the identified DEGs, most DEG transcription factors exhibited a trend of upregulation compared with those from the healthy fruits (Fig. 4).

C2H2 zinc finger proteins is a large gene family in plants that is involved in plant growth and developmen, so as biological and abiotic stress responses. After HSVd infection, the expressions of 19 genes were altered in this study. Most of them were upregulated.

The MYB family members are mainly involved in the plant hormone signal transduction, plant–pathogen interaction and anthocyanin biosynthesis. In this study, we observed that two MYB TFs were down-regulated and nine MYB TFs were up-regulated in the fruits of sweet cherry after HSVd infection.

The bHLH family has been characterized by its function in anthocyanin biosynthesis, phytochrome signaling and fruit dehiscence (Yang et al., 2017). Members of MYB/bHLH TFs or their complexes regulate distinct cellular processes such as responses to biotic stress, hormone signaling and cell death (Stracke, Werber & Weisshaar, 2001; Toledo-Ortiz, Huq & Quail, 2003). We observed the altered expression of 10 bHLH genes upon HSVd infection.

The AP2/ERF family has been shown to be involved in hormonal signaling, response to biological and abiotic stresses, and metabolism regulation (Nakano et al., 2006). We identified nine genes that were strongly affected by HSVd infection; seven of them were upregulated.

The Dof domain proteins act as transcriptional regulators in plant growth and development (Wen et al., 2016). Nine genes were altered by HSVd infection.

The NAC family members act as nodes of the regulatory network that responds to biotic stresses and also takes part in plant–pathogen interactions (Saidi, Mergby & Brini, 2017). We observed five NAC TFs that were affected by HSVd infection.

Members of the WRKY family are thought to be at the heart of the plant immune system and play an essential role in pathogen defense and plant hormone signaling (Eulgem & Somssich, 2007; Muthamilarasan et al., 2014, 2015). We identified four upregulated WRKY genes upon HSVd infection. Thus, WRKY TFs were also involved in HSVd-infected responses.

### Validation of RNA-seq data by RT-qPCR

**Figure 5:**
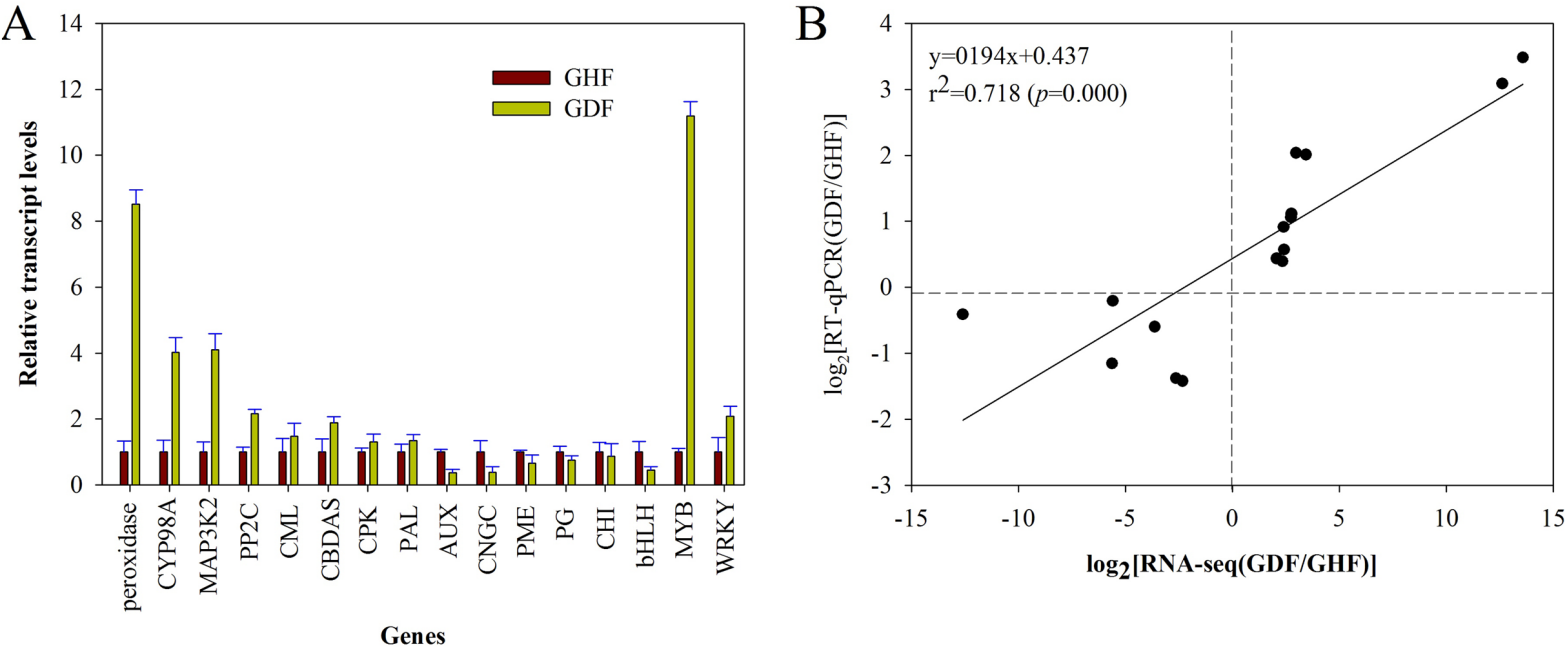
RT-qPCR validation of RNA-seq data of HSVd infected (GDF) and healthy (GHF) sweet cherry fruits. (A) Expression profiles of the selected genes as determined by RT-qPCR. (B) Scatter plot shows correaltion between trasncript level changes (log_2_ FC) measured by RNA-seq and by RT-qPCR analysis of the selected genes. Peroxidase:Peroxidase 43; CYP98A2: Cytochrome P450 98A2; MAP3K2: Mitogen-activated protein kinase kinase kinase 2; PP2C: Protein phosphatase 2C 24; CML: Calcium-binding protein CML45; CBDAS: Cannabidiolic acid synthase-like 1; CPK: Calciumdependent protein kinase 26; PAL: Phenylalanine ammonia-lyase 1; AUX: Auxin-induced protein AUX28-like; CNGC: Cyclic nucleotide-gated ion channel 15; PME: Probable pectinesterase/pectinesterase inhibitor 7; PG: Polygalacturonase 1; CHI: Chalcone isomerase; bHLH: Transcription factor bHLH79; MYB: Transcription factor MYB111; WRKY: WRKY transcription factor 70.

In order to validate the RNA-seq results, the gene expression changes of 16 randomly selected DEGs were analyzed by RT-qPCR with the primers listed in Table S1. The relative expression of these selected genes is shown in Fig. 5A. Moreover, the correlation between RNA-seq and RT-qPCR was measured by scatter plotting the log_2_-fold changes. A significant positive correlation between the results of RT-qPCR and RNA-Seq was obtained, suggesting that the results of RNA-Seq were reliable enough to discuss the effects of HSVd infection on the expression profiles (Fig. 5B; Table S1). These 16 genes exhibited consistent expression patterns between the RNA-Seq data and RT-qPCR.

## Discussion

HSVd has evolved a broad host range, including monocots and dicots, herbaceous and woody plants, which reflects its high potentiality of transmissibility (Kovalskaya & Hammond, 2014). Recently, it has been discovered in sweet cherry, causing severe dappled fruit symptoms, and has a great influence on exterior quality. RNA-Seq has been utilized to identify the transcriptomic changes that occured during HSVd infection in cucumber and hops (Xia et al., 2017; Kappagantu et al., 2017a). However, at present, there are no reports regarding the transcriptome responses of sweet cherry fruits to viroid infection. In this study, the transcriptome was sequenced to assess the differences in gene expressions in fruits of sweet cherry infected by HSVd. Compared with healthy, 1,572 DEGs were identified, of which 961 DEGs (61.13%) were upregulated and 611 DEGs (38.87%) were downregulated. These DEGs were mainly involved in plant–pathogen interactions, MAPK signaling pathway, plant hormone signal transduction pathways and secondary metabolism (phenylpropanoid biosynthesis and flavonoid biosynthesis). The pathways or genes could help us to understand the physiological and molecular mechanisms of HSVd infection in sweet cherry fruit and further develop disease prevention and treatment strategies.

During pathogen infection and disease progression, a number of defense pathways are triggered, such as salicylic acid (SA), jasmonic acid (JA) and abscisic acid (ABA), which are mainly regulated by hormone signaling pathways (Owens et al., 2012; Alazem & Lin, 2015; Rigal, Ma & Robert, 2014). The RNA-seq data showed that HSVd infection affected several genes that were associated with hormone signal transduction pathways. This indicates that most of the plant hormone signaling pathways were altered after HSVd infection of sweet cherry fruits. The genes involved in JA, SA, ABA and ET were upregulated in sweet cherry fruits. Auxin promotes the degradation of a family of transcriptional repressors known as auxin/indole-3-acetic acid (Aux/IAA) proteins that bind to auxin response factors and inhibit the expression of specific auxin response genes. Sun et al. (2019) conducted a genome-wide identification and comprehensive phylogenetic analysis of auxin early response genes in upland cotton, including Aux/IAA, GH3 and SAUR family members, which acted as a key component of auxin signaling. In the present study, the expression levels of *Aux1* and *GH3* were upregulated, and the expression level of *Aux/IAA* was downregulated. Moreover, six *SAUR* homologs were upregulated and five were downregulated. The results indicated that auxin early response genes were widely involved and played an important role in the response to HSVd infection. However, changes in the expression of genes that were associated with brassinosteroid (BA) and gibberellin (GA) metabolism or response were not observed in the present study. Similarly, genes involved in SA, JA, ET and ABA biosynthesis and responses were observed in CBCVd-infected hops and CEVd-infected tomato (Mishra et al., 2018), and no significantly different genes were associated with BA-or GA-dependent pathways in CEVd-infected tomato (Thibaut & Claude, 2018).

*Hop stunt viroid* infection disrupts phytohormone homeostasis and triggered basal defense responses. In plants, PTI and ETI constitute the front line of defense against pathogens (Macho & Zipfel, 2014). PTI or ETI are triggered by the activation of various membrane-associated receptor-like kinases (immune receptor) upon the perception of non-self components of pathogenic origins via MAPK and CDPKs signaling cascades that act downstream of receptor complexes (Mishra et al., 2018). The MAPK cascade plays a key role in signal transduction of PTI and ETI pathways and activates defense-related genes and TFs. Our data showed the high upregulation of genes encoding MAP3K2, as well as *CaM4*, *RBOH*, *PP2C*, *HMA*, *EIN*2 and *ACS*6 in the MAPK signaling pathway, which is consistent with the results of previous studies on (*Potato spindle tuber viroid*) PSTVd infection and (*Citrus exocortis viroid*) CEVd infection in tomato (Zheng et al., 2017; Thibaut & Claude, 2018). Besides these MAP kinases, the plant defense response is often associated with ion exchange, the generation of ROS and cell wall reinforcement. CNGCs, members of the superfamily of nonselective cation channels with six transmembrane domains play a critical role in diverse physiological functions and have an isoleucine glutamine (IQ) domain (Falcone Ferreyra et al., 2013; Urquhart et al., 2011). In this study two CNGCs were identified, and one upregulated and one downregulated after HSVd infection. When pathogen invasion occurs, CNGCs may be associated with signal transduction cascades that raise Ca^2+^ concentrations in the cytosol and activate CaMs and/or CaM-like proteins (CMLs) (Ali et al., 2007). The regulation of Ca^2+^ is one of the prime functions in plant innate immunity (Heo et al., 1999; Yamakawa et al., 2001). CaM and calmodulin-like (CML) proteins are major Ca^2+^ sensors, playing critical roles in the Ca^2+^ signaling pathway and serve as key regulators of pathogen-induced changes in gene expression in plant immune responses (Zeng et al., 2015). CDPKs are involved in the phosphorylation of the substrates of PTI and ETI pathways and play a critical role in phytohormone signaling pathways, thus influencing plant growth, development and stress responses (Gao, Cox & He, 2014; Xu & Huang, 2017). Our results showed three upregulated CaMs and one upregulated CDPK after HSVd infection, suggesting that these CaMs and CDPK were induced by HSVd. These genes might play significant roles in the response of sweet cherry fruits to HSVd infection.

Plant secondary metabolites are well known to be of importance in plant growth and development, and they can also function as defense molecules, protecting plants under various adverse conditions (Yang et al., 2012; Patra et al., 2013). In the present study, numerous genes involved in the secondary metabolism pathway were highly expressed, such as those involved in phenylpropanoid biosynthesis and flavonoid biosynthesis, which correspond to the significantly enriched pathways in KEGG analysis. Studies have shown that the phenylpropanoid biosynthesis pathway can generate secondary metabolites such as lignin, flavonoids and anthocyanins, which have been reported to play key roles in the resistance of plants to pathogen infection (Falcone Ferreyra, Rius & Casati, 2012; Shinde et al., 2017). Flavonoids are a large group of phenolic secondary metabolites that are widely present in plants and have been shown to posess diverse biological functions, such as flower and fruit pigmentation, as well as defenses against biotic and abiotic stresses (Falcone Ferreyra, Rius & Casati, 2012). In the present study, several genes related to general prenylflavonoids and flavonoids were influenced by HSVd infection in sweet cherry fruits. PAL serves as the first enzyme that is involved in the biosynthesis of secondary metabolites such as flavonoids, anthocyanins, and prenylflavonoids (Matoušek et al., 2016). We observed that one PAL was upregulated in this study.

Peroxidases atalyze the oxidation of substrates in the presence of hydrogen peroxide or anorganic peroxide, which are known to be induced in host plant tissues by a wide range of plant pathogens (Hernández et al., 2016; Gadea et al., 1996; Rizza et al., 2012). Our results suggested that three peroxidases were upregulated and one peroxidase was downregulated. Depending on the interaction between plants and viroids, different peroxidases may be up-and/or downregulated, which is in agreement with results on citrus and tomato plants infected by CEVd (Gadea et al., 1996; Rizza et al., 2012). Moreover, the coloration of the fruit is an important aspect for all fruiting plants, mainly due to the presence of flavonoids. The flavonoids are made up mainly of anthocyanins, flavones, chalcones, flavanones, flavonols and isoflavonoids. Anthocyanins are responsible for most pinks, reds, mauves and blues in flowers and fruits (Han et al., 2010).

Previous studies have shown that anthocyanin biosynthesis required PAL, CHS, CHI, F3′H, F3′5′H, DFR, ANS and other enzymes (Koes et al., 1989; Bogs et al., 2006). In the present study, we observed that many genes associated with phenylpropanoid and flavonoid pathways, such as *PAL*, *CHS*, *CHI*, *FLS* and *HST*, were significantly altered by HSVd infection, which might be correlated to symptom (dapple) development in HSVd-infected sweet cherry fruits. It is presumed that disease symptoms may be the result of viroid-induced disruptions of secondary metabolism in host plants.

It is known that TFs play a pivotal role in plant growth and development and also respond to biotic and abiotic stimuli (Zhang, Lubberstedt & Xu, 2013; Mickelbart, Hasegawa & Bailey-Serres, 2015; Alves et al., 2014; Li et al., 2017; Sun, Fan & He, 2019). The transcription factors such as the C2H2 zinc finger, bZIP (basic domain/leucine zipper), WRKY, AP2 (APETALA2)/ERF (ethylene-responsive factor), NAM/ATAF/CUC(NAC), MYB, MYC and bHLH (basic helix–loop–helix), were significantly altered after viroid infection in plants (Wiesyk et al., 2018). Similarly, in the present study, 96 differentially expressed TFs were identified, with the highest number annotated in the following families: C2H2 zinc finger, followed by MYB, bHLH, AP2/ERF, C2C2-Dof, NAC and WRKY, which further strengthened their regulatory roles in defense responses against viroid infection in plants. These studies have opened new avenues for exploring and exploiting TFs to improve disease resistance against viroids in plants.

## Conclusions

In this study, we used RNA sequencing technology to analyze the gene expression profiles of sweet cherry fruits infected by HSVd. The results indicated that HSVd infection affected the gene expression levels of 1,572 DEGs, involving 961 upregulated and 611 downregulated DEGs. According to the analyses of GO and KEGG pathways, functional analysis of DEGs showed that these DEGs were mainly involved in plant hormone signal transduction, plant–pathogen interactions and the MAPK signaling pathway. HSVd infection also affected the secondary metabolism, such as both phenylpropanoid and flavonoid biosyntheses, which might be directly related to the development of symptoms in sweet cherry fruits infected by HSVd. Further research should focus on whether and how these pigmentation-related genes play an important role in fruit coloration. The transcript levels of several TF families also were affected, including the C2H2 zinc finger, MYB, bHLH, AP2/ERF, C2C2-Dof, NAC and WRKY. In conclusion, the analysis of the differential expression of HSVd-infected sweet cherry fruits will help to investigate the detailed mechanisms of plant responses to viroid infection and will contribute to a better understanding of the uderlying molecular and biochemical processes in order to develope strategies for combating viroid diseases in sweet cherry production.

## Supplemental Information

10.7717/peerj.10005/supp-1 Supplemental Information 1 Specific primer sequences of 16 selected genes used for RT-qPCR validation of RNA-seq and the correlation between RT-qPCR and RNA-seq. Gene ID: gene designation number; GDF: green Dapple Fruit; GHF: green Healthy Fruit.

10.7717/peerj.10005/supp-2 Supplemental Information 2 Annotation of DEGs of HSVd-responsive sweet cherry fruits transcriptome. Gene ID: gene designation number; fpkm: expected number of Fragments Per Kilobase of transcript sequence per Millions base pairs sequenced; *p*-value: statistical significance test index; padj: the corrected *p*-value; COG: cluster of Orthologous Groups of proteins; GO: gene Ontology; KEGG: kyoto Encyclopedia of Genes and Genomes; nr: refSeq non-redundant proteins.

10.7717/peerj.10005/supp-3 Supplemental Information 3 GO term enrichment for differentially expressed genes. BP: Biological Process; CC: Cellular Component; p-value: Statistical significance test index; padj: The corrected p-value.

10.7717/peerj.10005/supp-4 Supplemental Information 4 Differentially expressed genes in the KEGG pathways. KEGG: Kyoto Encyclopedia of Genes and Genomes; p-value: Statistical significance test index; padj: The corrected p-value

10.7717/peerj.10005/supp-5 Supplemental Information 5 Differentially expressed genes in plant hormone signal transduction pathway. GDF: Green Dapple Fruit; GHF: Green Healthy Fruit; fpkm: Expected number of Fragments Per Kilobase of transcript sequence per Millions base pairs sequenced; p-value: Statistical significance test index; padj: The corrected p-value; nr: RefSeq non-redundant proteins; KEGG: Kyoto Encyclopedia of Genes and Genomes.

10.7717/peerj.10005/supp-6 Supplemental Information 6 Differentially expressed genes in basal defense responses. GDF: Green Dapple Fruit; GHF: Green Healthy Fruit; fpkm: Expected number of Fragments Per Kilobase of transcript sequence per Millions base pairs sequenced; p-value: Statistical significance test index; padj: The corrected p-value; nr: RefSeq non-redundant proteins; KEGG: Kyoto Encyclopedia of Genes and Genomes

10.7717/peerj.10005/supp-7 Supplemental Information 7 Differentially expressed genes in secondary metabolites metabolism. GDF: Green Dapple Fruit; GHF: Green Healthy Fruit; fpkm: Expected number of Fragments Per Kilobase of transcript sequence per Millions base pairs sequenced; p-value: Statistical significance test index; padj: The corrected p-value; nr: RefSeq non-redundant proteins; KEGG: Kyoto Encyclopedia of Genes and Genomes

10.7717/peerj.10005/supp-8 Supplemental Information 8 Transcription factors involved in responses to HSVd infection in sweet cherry fruits. GDF: Green Dapple Fruit; GHF: Green Healthy Fruit;fpkm: Expected number of Fragments Per Kilobase of transcript sequence per Millions base pairs sequenced; *p*-value: Statistical significance test index; padj: The corrected *p*-value; nr: RefSeq non-redundant proteins; KEGG: Kyoto Encyclopedia of Genes and Genomes.

10.7717/peerj.10005/supp-9 Supplemental Information 9 The raw data of RT-qPCR.

## Additional Information and Declarations

### Data Availability

The following information was supplied regarding data availability: The data is available at SRA: PRJNA596893.
